# Gut Microbiota Differs Between Parkinson’s Disease Patients and Healthy Controls in Northeast China

**DOI:** 10.3389/fnmol.2019.00171

**Published:** 2019-07-11

**Authors:** Chunxiao Li, Li Cui, Yimin Yang, Jing Miao, Xiuzhen Zhao, Jingdian Zhang, Guohong Cui, Ying Zhang

**Affiliations:** ^1^Department of Neurology and Neuroscience Center, The First Hospital of Jilin University, Changchun, China; ^2^Department of Intensive Care Unit, First Hospital of Jilin University, Changchun, China; ^3^Neurobiology Laboratory, National Institute of Environmental Health Sciences, National Institutes of Health, Durham, NC, United States

**Keywords:** Parkinson’s disease, gut microbiota, dysbiosis, *Akkermansia*, *Lactobacillus*

## Abstract

**Background**: There is accumulating evidence suggesting a connection between the gut and Parkinson’s disease (PD). Gut microbiota may play an important role in the intestinal lesions in PD patients.

**Objective**: This study aims to determine whether gut microbiota differs between PD patients and healthy controls in Northeast of China, and to identify the factors that influence the changes in the gut microbiota.

**Methods**: We enrolled 51 PD patients and 48 healthy controls in this study. Microbial species in stool samples were determined through 16S-rRNA gene sequencing. Dietary intakes were collected from a subset of 42 patients and 23 controls using a food frequency questionnaire (FFQ). Gut microbiota species richness, diversity, differential abundance of individual taxa between PD patients and controls, and the relationship between the gut microbiota abundance and the dietary and clinical factors were analyzed.

**Results**: PD patients showed decreased species richness, phylogenetic diversity, β- diversity, and altered relative abundance in several taxa compared to the controls. PD- associated clinical scores appeared to be the most influential factors that correlated with the abundance of a variety of taxa. The most consistent findings suggested by multiple analyses used in this study were the increase of *Akkermansia* and the decrease of *Lactobacillus* in PD patients in Northeast China.

**Conclusion**: Gut microbiota significantly differed between a group of PD patients and healthy controls in Northeast China, with decreased species richness, phylogenetic diversity, β-diversity, and altered relative abundance in several taxa compared to the controls.

## Background

Parkinson’s disease (PD) is a common neurodegenerative disorder characterized by motor and non-motor symptoms. The motor symptoms include bradykinesia, resting tremor, rigidity, and postural instability (Reichmann, 2010). These symptoms are mainly attributable to the degeneration and loss of dopaminergic neurons in the substantia nigra of the midbrain (Chung et al., 2001). In contrast, the non-motor symptoms of PD, such as constipation, hyposmia, depression, and cognitive impairment (Chen et al., 2013) cannot be explained by the changes in the substantia nigra. Braak et al. (2003) found that in PD, pathological changes were not limited to the substantia nigra, but rather involved multiple parts of the nervous system. In the pre-symptomatic stage, pathological changes are confined to the medulla oblongata/pontine tegmentum and olfactory bulb/anterior olfactory nucleus. As the disease progresses, the substantia nigra and other nuclei in the midbrain and forebrain become the main areas of pathological involvement. Eventually, the pathological changes spread to the neocortex (Braak et al., 2004) causing cognitive symptoms. Recently, the non-motor symptoms of PD have become a focus of PD studies. One such symptom is constipation, which is common and often precedes the motor symptoms (Cersosimo et al., 2013). Lewy bodies and α-synuclein, which are the neuropathological hallmarks of PD, may appear in the gut before they appear in the brain (Adler and Beach, 2016). In PD patients, constipation is often associated with α-synuclein accumulation and neurodegeneration in the enteric nervous system (Cersosimo and Benarroch, 2012), with local inflammation, oxidative stress, and increased intestinal permeability (Forsyth et al., 2011; Devos et al., 2013). These pathophysiological changes occur at the initial stages of PD, and in some cases, may predate the motor symptoms by years (Savica et al., 2009; Shannon et al., 2012). Therefore, it is speculated that lesion in the gut may be one of the triggering factors that contribute to the pathogenesis of PD.

The gut microbiota is an important component of the intestinal environment. It can affect immune function and development (Hooper et al., 2012), metabolism (Sonnenburg and Bäckhed, 2016), as well as the central nervous system (Mayer et al., 2014). Several psychiatric and neurological disorders have been found to be associated with changes in the gut microbiota, including schizophrenia (Severance et al., 2016), depression (Zheng et al., 2016), autism (Kang et al., 2013), multiple sclerosis (Berer et al., 2011; Lee et al., 2011) and Alzheimer’s disease (Minter et al., 2017). Recently, many studies have shown that the composition of gut microbiota significantly differs between PD patients and control subjects, but the results varied widely among these studies (Hasegawa et al., 2015; Keshavarzian et al., 2015; Mulak and Bonaz, 2015; Scheperjans et al., 2015; Felice et al., 2016; Unger et al., 2016; Hill-Burns et al., 2017; Li et al., 2017). However, this is not altogether surprising, as the gut microbiota is affected by many factors, such as lifestyle, diet (Smits et al., 2017), age, geography (Yatsunenko et al., 2012), body mass index (BMI), and race (Chen et al., 2016). In this study, we aim to determine whether gut microbiota profiles differ between PD patients and control subjects living in the northeast region of China.

## Materials and Methods

### Subjects

We enrolled 51 patients who had been diagnosed with PD according to the diagnostic criteria proposed by the International Parkinson Disease and Movement Disorder Society in 2015 (Postuma et al., 2015) in the First Hospital of Jilin University. To be included in the study, PD patients were required to meet the following criteria: no antibiotic use for at least 3 months prior to the study; no digestive system diseases, such as inflammatory bowel disease, no diseases affecting the liver, gall bladder, or pancreas, and no history of surgery on the digestive tract; non-smoking; no alcohol consumption for at least 2 years prior to the study; no autoimmune disease, such as diabetes; no family history of PD; and age at PD onset >50 years. The patients were on PD medications including carbidopa/levodopa and dopamine agonists when the samples were collected. Subjects with a history of using medications that have been shown to affect gut microbiota, including COMT inhibitors, anticholinergics, anti-secretory drugs, or cardiological drugs within the 3 months before the start of the study were excluded.

We also recruited the spouses of the PD patients as healthy controls (*n* = 39) to minimize the influence of lifestyle factors on the gut microbiota. Recruiting the spouses of PD patients as controls could also help offsetting the potential impact on gut microbiota by the physical and socioeconomic stress that is present in both PD patients and their spouses (O’Reilly et al., 1996; Hemmerle et al., 2012; Anderson et al., 2016). To ensure a sufficient sample size, we further recruited nine age-matched, healthy controls from the local community. Thus, in total, we recruited 48 healthy controls who met the following criteria: no neurodegenerative diseases; no digestive system diseases, no diseases affecting the liver, gall bladder, or pancreas, and no history of surgery on the digestive tract; no hypertension, diabetes, or immune diseases; no antibiotic use, no proton pump inhibitors use, no cardiological drugs use for at least 3 months prior to the study; non-smoking; and no alcohol consumption for more at least 2 years prior to the study (Table 1). All subjects were of Han Chinese ethnicity and resided in the northeast region of China. In addition, patients and controls were well-matched in terms of age and BMI.

**Table 1:** Demographic characteristics of all the participants.

Demographic factor	PD	Control	*P*-value^#^
Total subjects	51	48	NA
Male	32	19	NA
Female	19	29	NA
Age (years, mean ± SD)	62.4 ± 8.2	62.2 ± 9.2	0.746
BMI (kg/m2, mean ± SD)	24.9 ± 3.8	24.5 ± 2.6	0.711
PD duration (years, mean ± SD)	4.55 ± 3.59	NA	NA

*^#^Unpaired *t*-test; PD, Parkinson’ disease; SD, standard deviation; BMI, body mass index*.

### Clinical Data Collection

Parkinsonian motor symptoms were measured using the Movement Disorder Society Unified Parkinson Disease Rating Scale (UPDRS)-III (Movement Disorder Society Task Force on Rating Scales for Parkinson’s Disease, 2003) and the modified Hoehn and Yahr scale (Goetz et al., 2004). Non-motor symptoms were assessed using the Non-Motor Symptom Questionnaire (NMSQ; Chaudhuri et al., 2006), Scales for Outcomes in Parkinson’s Disease—autonomic symptoms (SCOPA-AUT; Visser et al., 2004), and Rapid Eye Movement Sleep Behavior Disorder questionnaire—Hong Kong (RBDQ-HK) scale (Shen et al., 2014) in the “on” state. PD duration was defined as the interval between the patient’s age at the time of the study and their age at PD onset (Table 1). Constipation was measured according to the Wexner scoring scale (Agachan et al., 1996). Medication data were extracted from the patients’ prescriptions at the time of this study.

### Patient Groups and Subgroups

Our study population was divided into a PD group (*N* = 51) and a control group (*N* = 48). The PD group was further divided into subgroups according to the patients’ scores on various scales but resulted in too small sample sizes. Thus, the data were not further analyzed and were not presented in this study.

A subset of PD patients (*N* = 42) and controls (*N* = 23) completed a modified Chinese food frequency questionnaire (FFQ). The rest of the subjects only provided the stool samples but declined the request for further completing the questionnaires. We quantified their dietary fiber, carbohydrate, and fat intake according to Chinese food composition tables, and found no difference between the PD and control groups (Table 2, Supplementary Figure S1).

**Table 2:** PD characteristics, constipation score and dietary fiber intake information collected from a subset of the participants.

	PD	Control	*P*-value^#^
Number of subjects	42	23	NA
Modified Hoehn and Yahr scale	1.70 ± 0.73	NA	NA
UPDRS-III (mean ± SD)	24.55 ± 12.38	0.78 ± 0.74	*P* < 0.0001
NMSQ (mean ± SD)	12.07 ± 3.85	1.043 ± 0.88	*P* < 0.0001
SCOPA-AUT (mean ± SD)	16.74 ± 8.23	1 ± 0.60	*P* < 0.0001
RBDQ-HK (mean ± SD)	16.93 ± 14.32	2.44 ± 1.81	*P* < 0.0001
Constipation (Wexner	5.93 ± 4.05	0.85 ± 0.82	*P* < 0.0001
score, mean ± SD)			
Dietary fiber	12.56 ± 5.47	12.64 ± 3.74	*P* = 0.95
(g/day, mean ± SD)			

*^#^Unpaired *t*-test; PD, Parkinson’ disease; SD, standard deviation; UPDRS-III, The third part of the Unified Parkinson’s Disease Rating Scale; SCOPA-AUT, Scale for Outcomes in PD for Autonomic Symptoms; NMSQ, non-movement syndrome questionnaire; RBDQ-HK, REM Sleep Behavior Disorder Questionnaire-Hong Kong*.

### Stool Collection

Stool samples were collected at home in sterile, sealed tubes by the subjects themselves, according to our instructions. The subjects were told to pass stools in a clean basin, place the middle section of the stools into a sterilized tube, and store the samples at −20°C in a freezer. Our researchers collected the stool samples within 3 days, cooled them with liquid nitrogen, and stored them at −80°C until analysis.

### 16S rRNA Amplicon Analysis

DNA extraction from the stool samples and 16S rRNA amplicon sequencing were performed using the HiSeq2500 PE250 sequencing platform, according to the manufacturer’s instructions. Briefly, fecal DNA was extracted using QIAamp Fast DNA Stool Mini Kit (Qiagen) and diluted to 1 ng/μl before PCR amplification. Barcoded primers (515F and 806R) were used to target the V4 region of the 16S rRNA gene. Amplicons were purified and sequenced using HiSeq2500 PE250 sequencing platform. Raw tags were formed by linking the reads from each sample using FLASH (V1.2.7^1^) and filtered to obtain high quality clean tags according to published protocols (Qiime V1.7.0^2^). The final effective tags were obtained by removing the chimera sequence from clean tags (Caporaso et al., 2010; Edgar et al., 2011; Haas et al., 2011; Magoč and Salzberg, 2011; Bokulich et al., 2013). All samples were sequenced in the same laboratory. Operational taxonomic units (OTUs) were selected using Uparse v7.0.1001 (Edgar, 2013) at 97% similarity for all effective tags. The representative sequence of the OTUs was analyzed using the mothur program and SILVA SSU rRNA database (Wang et al., 2007; Quast et al., 2013).

### Species Richness and Alpha-Diversity

We analyzed within-community microbial diversity by using α-diversity analysis (Li et al., 2013). We calculated the Observed-species, Chao1, Shannon, Simpson, ACE, Goods-coverage, and PD_whole_tree indexes by using Qiime v1.7.0. We plotted the rarefaction curve, rank abundance curve (Lundberg et al., 2013), and species accumulation boxplot with R v2.15.3. We analyzed the differences in these indices between the PD and control groups by using *t*-test and Wilcoxon rank-sum test.

### Beta-Diversity

We calculated the dissimilarities (distance) in the microbiomes, including unweighted unique fraction metric (Unifrac; Lozupone and Knight, 2005; Lozupone et al., 2011) and weighted Unifrac (Lozupone et al., 2007), between the PD and control groups by using the unweighted pair-group method with arithmetic mean (UPGMA) by Qiime software (Version 1.7.0). We performed principal co-ordinates analysis by using the WGCNA, stats, and ggplot2 packages of the R software. Analysis of similarities (Anosim), multi-response permutation procedures (MRPP) analysis, and Adonis (permutational multivariate analysis of variance; Zapala and Schork, 2006) were conducted using the R software. Differences in the weighted and unweighted Unifrac between the PD and control groups were analyzed using the *t*-test and Wilcoxon rank-sum test by the R software.

### Differential Abundance Analysis of Gut Microbiota Between PD Patients and Controls

Linear discriminant analysis (LDA) effect size (LEfSe) analysis (Segata et al., 2011) was conducted using the LEfSe software, and the threshold for LDA score was set at 4. Permutation tests were conducted between groups at various classification levels (phylum, class, order, family, genus, and species) by using the R software, and *P*-values were obtained. The *P*-values were modified to *q*-values (White et al., 2009) by using the Benjamini and Hochberg false discovery rate method. Differences between groups were also analyzed using the *t*-test and Wilcoxon rank-sum test in R software.

### Correlations Between Microbiome Abundance and Clinical and Dietary Factors

To elucidate the potential relationship between the gut microbiota and clinical factors such as the UPDRS, NMSQ, and SCOPA scores and dietary factors, we conducted a Spearman correlation analysis to study the relationship between the clinical and dietary factors mentioned above and gut microbiota abundance, focusing on bacteria with significant differences between PD and control groups identified in the *t*-test analysis.

#### Quantification of the Effect Size

To quantify the effect size in the *t*-test analyses, we calculated the Hedge’s g. To quantify the effect size for the Wilcoxon rank-sum test, we calculated the rank biserial correlation coefficient r.

## Results

### The Differences of Gut Microbiota Profiles Between PD and Control Groups

**Figure 1:**
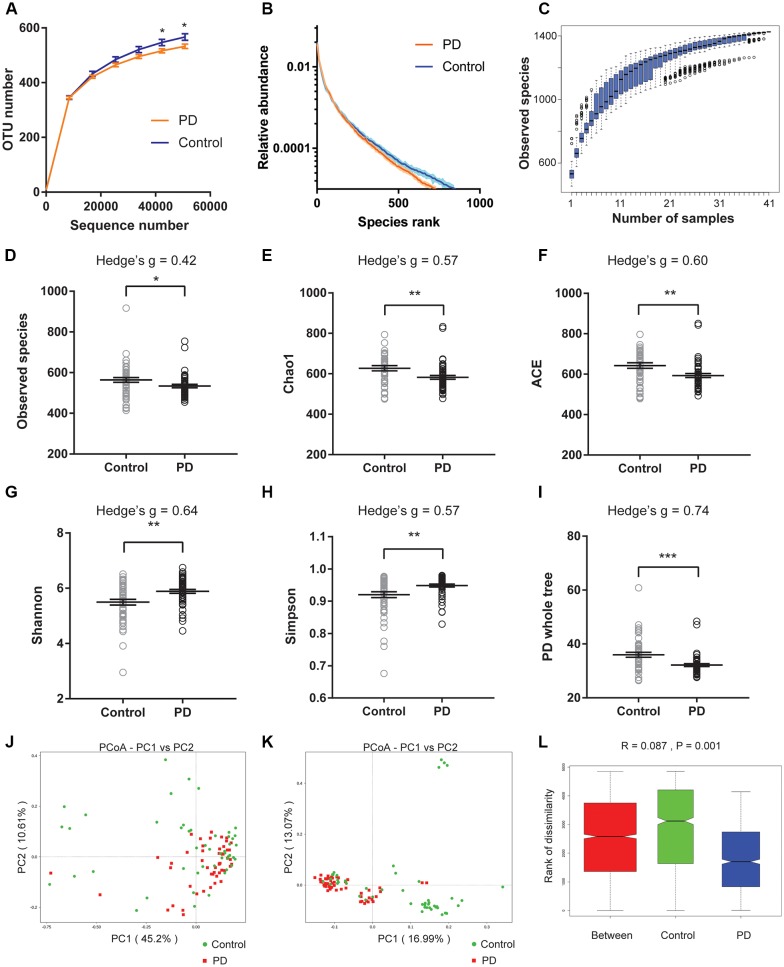
Altered gut microbiota richness and diversity indices in Parkinson’s disease (PD) patients. **(A)** Rarefaction curves to show the adequate depth of the sequencing. Data are presented as mean ± standard error of the mean (SEM). *n* = 51 for PD, *n* = 48 for control. * *p* < 0.05, unpaired *t*-test. **(B)** Rank abundance curve to show the relative species abundance of the samples from PD patients and controls. Data are presented as mean ± SEM. **(C)** Species accumulation boxplot to show that the number of samples is also adequate. **(D–I)** Comparison of species richness **(D–F)**, α-diversity **(G–H)**, and phylogenetic diversity **(I)** indices between PD patients and controls (*n* = 51 for PD, *n* = 48 for control). Data are presented as mean ± SEM with individual replicates also shown. *p < 0.05, **p < 0.01, ***p < 0.001, unpaired *t*-test. **(J–L)** β-diversity analyses using Principal Co-ordinates Analysis (PCoA) plotted with weighted Unifrac **(J)** and unweighted Unifrac **(K)**, and Analysis of similarities (ANOSIM; **L**).

We first plotted the rarefaction curves to show that they approached a plateau, indicating that the sequencing depth was adequate in this study (Figure 1A). We also plotted the rank abundance curves to compare the richness and evenness between PD and control groups and found reduced richness (less spread on x-axis) and evenness (steeper slope) in PD patients (Figure 1B). We further plotted species cumulation boxplot to confirm that the sample size of our study is adequate for valid analysis (Figure 1C).

We next calculated the indices to compare the richness (‘Observed species’, Chao1, ACE, Figures 1D–F), α-diversity (Shannon, Simpson, Figures 1G,H), and phylogenetic diversity (‘PD whole tree’, Figure 1I) between PD and controls of all recruited subjects (Figures 1D–I), and between PD and controls with no or mild constipation (defined as Wexner score ≤ 3, Supplementary Figure S2). The obtained indices suggested decreased species richness and phylogenetic diversity, and increased α-diversity in PD patients when all subjects were included in the comparisons. However, the α-diversity indices (Shannon and Simpson) were not significantly different between PD patients and controls when only the subjects with Wexner score ≤ 3 were compared, suggesting that constipation could be a confounding factor that impacts the α-diversity (Zhu et al., 2014; Mancabelli et al., 2017) or due to reduced sample size in each group. To further rule out the potential impact of gastric distress on these results, we carried out correlation analyses to investigate the relationship between Wexner scores and the gut microbiota richness and diversity indices (Supplementary Figures S3A–F). The correlations between the measured indices and the Wexner constipation score are weak, suggesting that the severity of constipation does not have a strong effect on these indices. To investigate the potential impact of gender on these findings, we performed the two-way ANOVA and did not find the significant main effect of gender (Supplementary Figure S4).

We then calculated weighted and unweighted Unifrac, and compared the β-diversity between PD patients and controls. Principal Co-ordinates Analysis (PCoA) plots showed more clustered distribution of the samples from PD patients compared to the samples from the controls (Figures 1J,K, Supplementary Figures S3G,H). Anosim analysis also revealed reduced distances between PD samples than the distances between the control samples (Figure 1L). Both analyses suggested reduced β-diversity in PD patients, which was further supported by MRPP analysis (*A* = 0.013, observed Δ = 0.559, expected Δ = 0.566, *p* = 0.001) and Adonis analysis (*R*^2^ = 0.035, *p* = 0.001).

**Figure 2:**
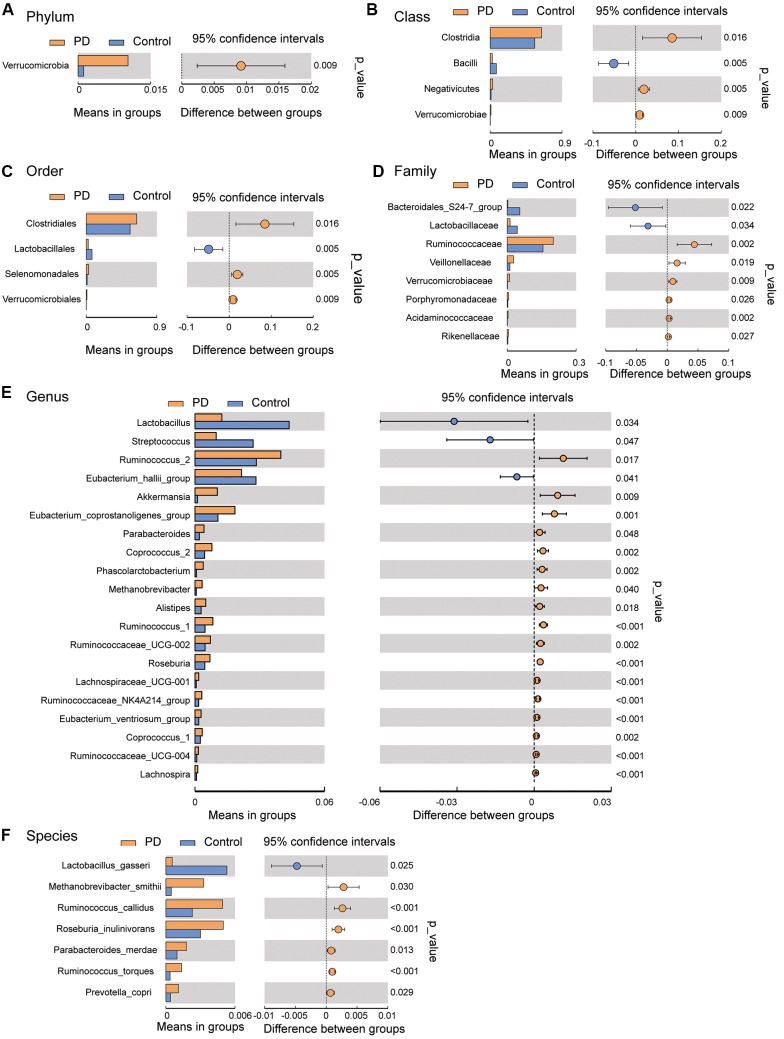
Gut microbiota differences between PD patients and controls detected by *t*-tests. Gut microbiota is compared between PD patients and healthy control subjects at phylum **(A)**, class **(B)**, order **(C)**, family **(D)**, genus **(E)**, and species **(F)** levels. Only the taxa with statistically significant difference are plotted. The bars on the left side of each figure show the relative abundance. On the right side of each figure, the center of circles represents the difference between the means of the two groups. The error bars represent the 95% confidence interval. *P*-values of unpaired *t*-test are listed on the right (*n* = 51 for PD, *n* = 48 for control).

**Figure 3:**
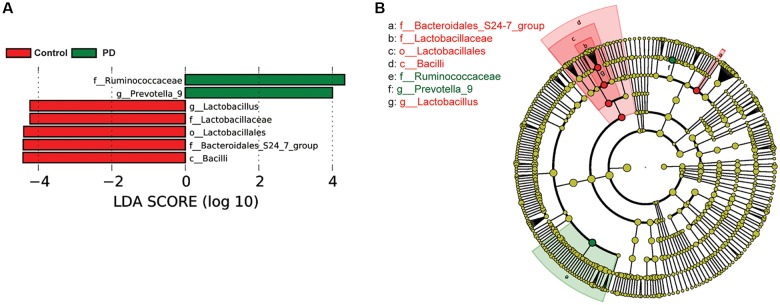
Gut microbiota differences between PD patients and controls detected by LEfSe analysis. **(A)** A bar graph of linear discriminant analysis (LDA) score distribution showing taxa with LDA scores greater than the set value of 4, that is, species that highly differ between the groups. **(B)** A cladogram to show the taxonomic structure and the relative abundance of the identified taxa. The size of each dot is proportional to the relative abundance of each taxon.

To investigate the differential abundance of individual taxa, we first ran *t*-tests of the relative abundance of each taxon between PD and controls at different phylogenetic ranks, and found significant difference in one phylum, four classes, four orders, eight families, 20 genera, and seven species (Figure 2). To verify these findings, we also performed the LEfSe analysis, which couples statistical significance with biological consistency and effect relevance. With LDA threshold set at 4, we identified one class, four orders, three families, and two genera that showed significant different abundances between the PD and control groups (Figure 3). The taxa identified by LEfSe were among the taxa that showed the most significant difference in the *t*-tests, except for *Prevotella_9*, which was not significant in the *t*-test.

To examine if constipation was a confounding factor that caused the differences in abundance of the taxa identified by the LEfSe analysis, we compared the relative abundance of these taxa between a subset of PD patients and controls with Wexner scores smaller than 3 using Wilcoxon rank-sum test (Supplementary Figure S5). We found that *Bacilli, Lactobacillales, Bacteroidales_S24–7_group, Lactobacillaceae and Lactobacillus* remained significantly different between PD and controls, whereas *Ruminococcaceae* and *Prevotella_9* lost significance. To further rule out the potential impact of gastric distress on these results, we carried out correlation analyses to investigate the relationship between Wexner scores and the individual taxa (Supplementary Figure S6). The correlations between the abundances and the Wexner constipation scores are weak, suggesting that the severity of constipation does not have a strong effect on these measurements. To examine the potential impact of gender on these findings, we again performed two-way ANOVA analysis to test the main effect of gender and PD on the abundance of the taxa, and found that the main effect of PD, but not gender, was significant (Supplementary Figure S7).

### Relationship of Gut Microbiota With Clinical and Dietary Factors

**Figure 4:**
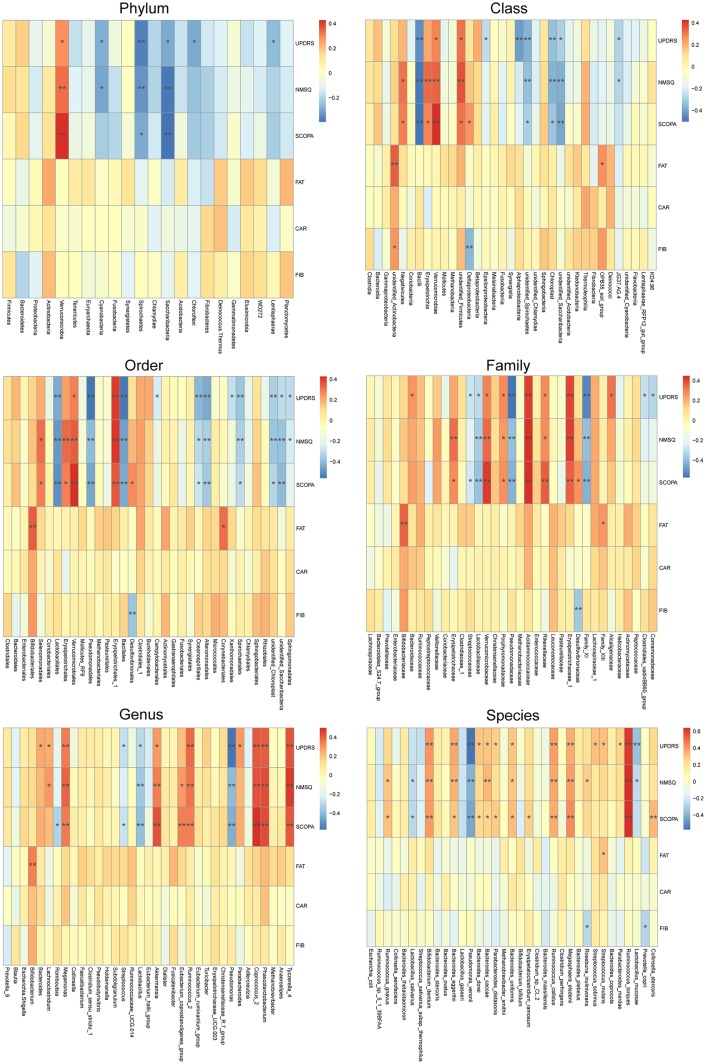
Correlations between clinical and dietary factors and gut microbiota when PD patients and controls were analyzed together. Correlation heatmaps showing the relationship between clinical and dietary factors and gut microbiota at different taxonomic ranks. Each row in the heatmap represents a clinical or dietary factor. Each column represents a taxon. The color temperature encodes Spearman correlation coefficient r. *n* = 65, *p < 0.05, ** *p* < 0.01.

**Figure 5:**
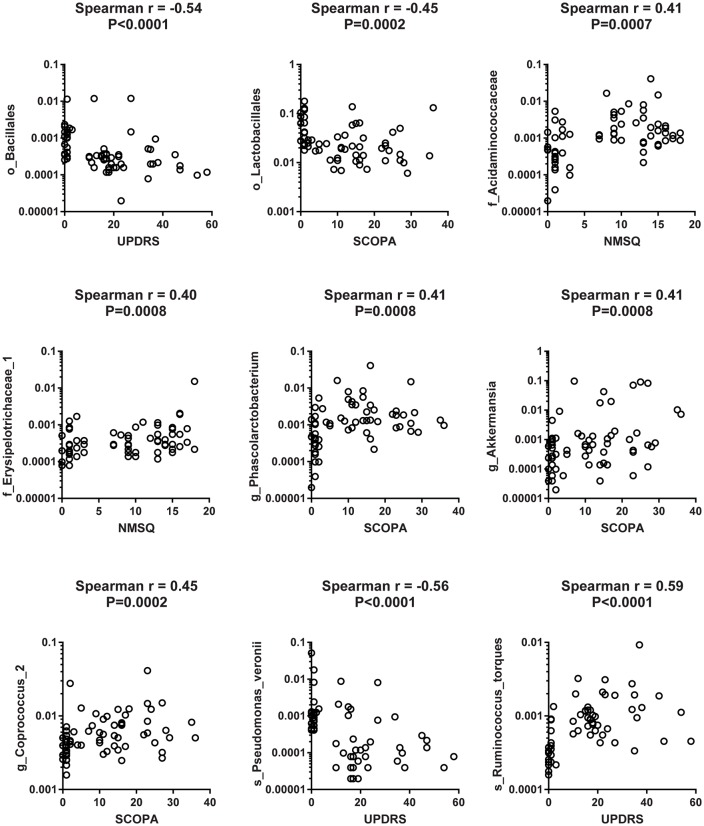
Scatter plots to show the strong correlation between clinical factors and gut microbiota when both PD patients and controls were included in the analysis. Examples of scatter plots to show that the clinical scores [Unified Parkinson Disease Rating Scale (UPDRS), Scales for Outcomes in Parkinson’s Disease (SCOPA) and Non-Motor Symptom Questionnaire (NMSQ)] were positively or negatively correlated with the abundance of specific taxa of gut microbiota at different taxonomic ranks when Spearman correlation analysis was performed on pooled data from 42 PD patients and 23 controls.

To reveal the relationships between clinical and dietary factors and the gut microbiota in control and PD patients, we performed Spearman correlation analysis to study the relationship between the clinical and dietary factors and gut microbiota abundance in 42 PD patients and 23 controls. We found that the PD-related clinical scores, such as UPDRS, NMSQ and SCOPA, were the most influential factors that either positively or negatively correlated with the abundance of the taxa at multiple phylogenetic ranks (Figure 4). The taxa that showed the strongest correlation with clinical factors included *Bacillales*, *Lactobacillales*, *Acidaminococcaceae*, *Erysipelotrichaceae_1*, *Phascolarctobacterium*, *Akkermansia*, *Coprococcus_2*, *Pseudomonas_veronii* and *Ruminococcus_torques* (Figure 5). We did not observe the strong correlations between clinical scores and the gut microbiota when the analysis was performed separately in PD patients (Supplementary Figure S8) or controls (Supplementary Figure S9), suggesting that the correlation we observed when all subjects were analyzed together more likely reflected the impact of PD as a disease condition, rather than being specific to individual clinical scores.

## Discussion

Our results revealed decreased species richness, phylogenetic diversity and β-diversity of gut microbiota in PD patients. More importantly, we identified several taxa that showed significant difference in abundance between PD patients and healthy controls using *t*-test and LEfSe analysis. Among these bacteria, some are particularly interesting because they have been linked to human health.

For example, *Akkermansia*, which was found higher in the gut of PD patients in our study (Figure 2E, consistent with previous findings; Keshavarzian et al., 2015; Unger et al., 2016; Hill-Burns et al., 2017), can degrade the colonic mucus barrier and increase pathogen susceptibility and intestinal permeability (Desai et al., 2016). Seregin et al. (2017b) have speculated that increased *Akkermansia* abundance may result in thinning of the intestinal mucus barrier, and allow for greater microbial access to the intestinal wall, which leads to the local inflammation driven by commensal organisms. In addition, *Akkermansia* abundance is negatively correlated with the innate immune receptor NLRP6 (Seregin et al., 2017b), the lack of which results in impaired interleukin-18 production and increased susceptibility to epithelial-induced injury (Chen et al., 2011; Elinav et al., 2011; Couturier-Maillard et al., 2013; Seregin et al., 2017a).

Another interesting finding of this study was the observed increase in the *Prevotella* genus and *Prevotella copri* species in PD patients. Increased abundance of *Prevotella copri* has been found in patients with rheumatoid arthritis (Scher et al., 2013) and HIV (Lozupone et al., 2014; Dillon et al., 2016), suggesting that it might be associated with inflammation. At the same time, *Prevotella* is involved in mucin synthesis in the gut mucosal layer, possibly contributing to the gut barrier function and the production of neuroactive short-chain fatty acids (SCFAs) through fiber fermentation (Arumugam et al., 2011). It has been recently reported that SCFAs can promote the α-synuclein-mediated neuroinflammation in a mouse model of PD (Sampson et al., 2016). Thus, the elevated gut *Prevotella* levels may play a role in worsening the PD in human patients. Our LEfSe analysis suggested that the abundance of *Prevotella_9* was significantly higher in PD patients than in controls (Figure 3). However, it was not significant in the Wilcoxon rank-sum test that compared its abundance between the PD patients and controls without severe constipation (Supplementary Figure S5). Furthermore, *t*-test comparisons (Figure 2E) and the two-way ANOVA (Supplementary Figure S3) assessing the effect of gender and PD on the abundance of *Prevotella_9* did not return statistical significance between PD and controls. *Prevotella copri*, a species within the *Prevotella* genus, showed significant increase in PD patients in *t*-test (Figure 2F). However, it was not identified as one of the significantly increased taxa in the LEfSe analysis when the threshold of LDA score was set at 4 (Figure 3). The possible increases in the *Prevotella* genus and *Prevotella copri* species in PD patients, though reported previously in a study with a large sample size (Hill-Burns et al., 2017), contrast with other studies (Hasegawa et al., 2015; Scheperjans et al., 2015; Unger et al., 2016; Bedarf et al., 2017; Petrov et al., 2017).

One of the most consistent findings in our study supported by both *t*-tests, LEfSe and the phylogenetic connection was the decreased abundance of *Lactobacillales/Lactobacillaceae/Lactobacillus* in PD patients. This observation is in accordance with another recent study in Chinese PD patients (Qian et al., 2018) and a study in Germany (Scheperjans et al., 2015), but contrasted with the findings in other studies (Hasegawa et al., 2015; Scheperjans et al., 2015; Hill-Burns et al., 2017; Hopfner et al., 2017; Petrov et al., 2017). These differences may be attributed to racial and regional differences in the PD patients and controls being studied. Local cuisines vary between countries and areas, and different diets lead to significantly different compositions of the gut microbiota (Faith et al., 2011; Rey et al., 2013; David et al., 2014). For example, a staple food regularly consumed in Northeast China, locally called Suan Cai (Sour Cabbage, very similar to sauerkraut) is enriched with *Lactobacillus*. Regular consumption of this type of food may elevate the gut *Lactobacillus* abundance in healthy controls, and manifest its decrease in PD patients in Northeast China.

Gut microbiota alterations have recently been reported in Chinese PD patients (Lin et al., 2018; Qian et al., 2018). Some of the findings in these two studies were consistent with our results (e.g., reduced abundance of *Lactobacillus* in PD patients; Qian et al., 2018), and some were not. This is not too surprising because these two reports were studying the patients from the Southwest (Lin et al., 2018) and Southeast (Qian et al., 2018) of China. Geographic factors, such as ethnicity and local cuisines vary widely across China.

Finally, our correlation analysis revealed strong correlations between clinical factors and the gut microbiota abundances when all subjects were included (Figures 4, 5), but not when the patients and controls were analyzed separately (Supplementary Figures S8, S9). This suggests that the observed correlations between clinical factors and gut taxa are more likely reflecting the impact of PD as a general disease condition, rather than the correlation between specific clinical scores and specific gut microbiomes.

The direction of change in the relative abundance of a few species reported in this study appears to be contradictory to some previous studies in PD patients from other regions. This could be attributed to region-specific factors affecting the gut microbiota of the recruited subjects, or the relatively limited sample size of this study. For example, we only had 23 controls from whom the clinical scores and dietary information were collected. Further assessments of PD patients in Northeast China with larger cohorts are needed to resolve any discrepancies. Another limitation of this study is that the PD patients included in this study were on levodopa/carbidopa treatment, which may have some impact on the gut microbiota (Hill-Burns et al., 2017).

## Conclusion

Gut microbiota significantly differed between a group of PD patients and healthy controls in Northeast China. PD patients showed decreased species richness, phylogenetic diversity and β-diversity. PD patients also showed altered relative abundance in several microbes compared to the controls. The most consistent finding revealed by both *t*-tests and LEfSe analysis was the reduction of the lactic acid-producing, potentially beneficial bacteria in PD patients at multiple phylogenetic ranks. A healthy diet or dietary supplements that promote the growth of *Lactobacillus* could potentially be beneficial in mitigating PD-related pathological changes in the gut.

## Ethics Statement

This study was carried out in accordance with the recommendations of the medical ethics committee of the First Hospital of Jilin University with written informed consent from all subjects. All subjects gave written informed consent in accordance with the Declaration of Helsinki. The protocol was approved by the the medical ethics committee of the First Hospital of Jilin University.

## Author Contributions

LC, GC and YZ conceived the study. CL and LC collected and analyzed the data. YY, JM, XZ, JZ and GC helped with data analysis. CL, LC and GC wrote the manuscript with the input from YZ.

## Conflict of Interest Statement

The authors declare that the research was conducted in the absence of any commercial or financial relationships that could be construed as a potential conflict of interest.

## Footnotes

^1^http://ccb.jhu.edu/software/FLASH/

^2^http://qiime.org/scripts/split_libraries_fastq.html

## Abbreviations

PD: Parkinson’s disease
OTUs: Operational taxonomic units
SD: standard deviation
BMI: body mass index
UPDRS-III: The third part of the Unified Parkinson’s Disease Rating Scale
SCOPA-AUT: Scale for Outcomes in PD for Autonomic Symptoms
NMSQ: Non-Motor Symptom Questionnaire
RBDQ-HK: REM Sleep Behavior Disorder Questionnaire-Hong Kong.

## References

[B1] Adler C. H. Beach T. G. (2016). Neuropathological basis of nonmotor manifestations of Parkinson’s disease. Mov. Disord. 31, 1114–1119. 10.1002/mds.26605 27030013 PMC4981515

[B2] Agachan F. Chen T. Pfeifer J. Reissman P. Wexner S. D. (1996). A constipation scoring system to simplify evaluation and management of constipated patients. Dis. Colon Rectum 39, 681–685. 10.1007/bf02056950 8646957

[B3] Anderson G. Seo M. Berk M. Carvalho A. F. Maes M. (2016). Gut permeability and microbiota in Parkinson’s disease: role of depression, tryptophan catabolites, oxidative and nitrosative stress and melatonergic pathways. Curr. Pharm. Des. 22, 6142–6151. 10.2174/1381612822666160906161513 27604608

[B4] Arumugam M. Raes J. Pelletier E. Le Paslier D. Yamada T. Mende D. R.. (2011). Enterotypes of the human gut microbiome. Nature 473, 174–180. 10.1038/nature09944 21508958 PMC3728647

[B5] Bedarf J. R. Hildebrand F. Coelho L. P. Sunagawa S. Bahram M. Goeser F.. (2017). Functional implications of microbial and viral gut metagenome changes in early stage L-DOPA-naive Parkinson’s disease patients. Genome Med. 9:39. 10.1186/s13073-017-0428-y 28449715 PMC5408370

[B6] Berer K. Mues M. Koutrolos M. Rasbi Z. A. Boziki M. Johner C.. (2011). Commensal microbiota and myelin autoantigen cooperate to trigger autoimmune demyelination. Nature 479, 538–541. 10.1038/nature10554 22031325

[B7] Bokulich N. A. Subramanian S. Faith J. J. Gevers D. Gordon J. I. Knight R.. (2013). Quality-filtering vastly improves diversity estimates from Illumina amplicon sequencing. Nat. Methods 10, 57–59. 10.1038/nmeth.2276 23202435 PMC3531572

[B8] Braak H. Del Tredici K. Rub U. de Vos R. A. Jansen Steur E. N. Braak E. (2003). Staging of brain pathology related to sporadic Parkinson’s disease. Neurobiol. Aging 24, 197–211. 10.1016/s0197-4580(02)00065-9 12498954

[B9] Braak H. Ghebremedhin E. Rub U. Bratzke H. Del Tredici K. (2004). Stages in the development of Parkinson’s disease-related pathology. Cell Tissue Res. 318, 121–134. 10.1007/s00441-004-0956-9 15338272

[B10] Caporaso J. G. Kuczynski J. Stombaugh J. Bittinger K. Bushman F. D. Costello E. K.. (2010). QIIME allows analysis of high-throughput community sequencing data. Nat. Methods 7, 335–336. 10.1038/nmeth.f.303 20383131 PMC3156573

[B11] Cersosimo M. G. Benarroch E. E. (2012). Pathological correlates of gastrointestinal dysfunction in Parkinson’s disease. Neurobiol. Dis. 46, 559–564. 10.1016/j.nbd.2011.10.014 22048068

[B12] Cersosimo M. G. Raina G. B. Pecci C. Pellene A. Calandra C. R. Gutierrez C.. (2013). Gastrointestinal manifestations in Parkinson’s disease: prevalence and occurrence before motor symptoms. J. Neurol. 260, 1332–1338. 10.1007/s00415-012-6801-2 23263478

[B13] Chaudhuri K. R. Martinez-Martin P. Schapira A. H. Stocchi F. Sethi K. Odin P.. (2006). International multicenter pilot study of the first comprehensive self-completed nonmotor symptoms questionnaire for Parkinson’s disease: the NMSQuest study. Mov. Disord. 21, 916–923. 10.1002/mds.20844 16547944

[B15] Chen H. Burton E. A. Ross G. W. Huang X. Savica R. Abbott R. D.. (2013). Research on the premotor symptoms of Parkinson’s disease: clinical and etiological implications. Environ. Health Perspect. 121, 1245–1252. 10.1289/ehp.1306967 23933572 PMC3855519

[B14] Chen G. Y. Liu M. Wang F. Bertin J. Nunez G. (2011). A functional role for Nlrp6 in intestinal inflammation and tumorigenesis. J. Immunol. 186, 7187–7194. 10.4049/jimmunol.1100412 21543645 PMC3133458

[B16] Chen J. Ryu E. Hathcock M. Ballman K. Chia N. Olson J. E.. (2016). Impact of demographics on human gut microbial diversity in a US Midwest population. PeerJ 4:e1514. 10.7717/peerj.1514 26839739 PMC4734456

[B17] Chung K. K. Zhang Y. Lim K. L. Tanaka Y. Huang H. Gao J.. (2001). Parkin ubiquitinates the α-synuclein-interacting protein, synphilin-1: implications for Lewy-body formation in Parkinson disease. Nat. Med. 7, 1144–1150. 10.1038/nm1001-1144 11590439

[B18] Couturier-Maillard A. Secher T. Rehman A. Normand S. De Arcangelis A. Haesler R.. (2013). NOD2-mediated dysbiosis predisposes mice to transmissible colitis and colorectal cancer. J. Clin. Invest. 123, 700–711. 10.1172/JCI62236 23281400 PMC3561825

[B19] David L. A. Maurice C. F. Carmody R. N. Gootenberg D. B. Button J. E. Wolfe B. E.. (2014). Diet rapidly and reproducibly alters the human gut microbiome. Nature 505, 559–563. 10.1038/nature12820 24336217 PMC3957428

[B20] Desai M. S. Seekatz A. M. Koropatkin N. M. Kamada N. Hickey C. A. Wolter M.. (2016). A dietary fiber-deprived gut microbiota degrades the colonic mucus barrier and enhances pathogen susceptibility. Cell 167, 1339.e21–1353.e21. 10.1016/j.cell.2016.10.043 27863247 PMC5131798

[B21] Devos D. Lebouvier T. Lardeux B. Biraud M. Rouaud T. Pouclet H.. (2013). Colonic inflammation in Parkinson’s disease. Neurobiol. Dis. 50, 42–48. 10.1016/j.nbd.2012.09.007 23017648

[B22] Dillon S. M. Lee E. J. Kotter C. V. Austin G. L. Gianella S. Siewe B.. (2016). Gut dendritic cell activation links an altered colonic microbiome to mucosal and systemic T-cell activation in untreated HIV-1 infection. Mucosal Immunol. 9, 24–37. 10.1038/mi.2015.33 25921339 PMC4626441

[B23] Edgar R. C. (2013). UPARSE: highly accurate OTU sequences from microbial amplicon reads. Nat. Methods 10, 996–998. 10.1038/nmeth.2604 23955772

[B24] Edgar R. C. Haas B. J. Clemente J. C. Quince C. Knight R. (2011). UCHIME improves sensitivity and speed of chimera detection. Bioinformatics 27, 2194–2200. 10.1093/bioinformatics/btr381 21700674 PMC3150044

[B25] Elinav E. Strowig T. Kau A. L. Henao-Mejia J. Thaiss C. A. Booth C. J.. (2011). NLRP6 inflammasome regulates colonic microbial ecology and risk for colitis. Cell 145, 745–757. 10.1016/j.cell.2011.04.022 21565393 PMC3140910

[B26] Faith J. J. McNulty N. P. Rey F. E. Gordon J. I. (2011). Predicting a human gut microbiota’s response to diet in gnotobiotic mice. Science 333, 101–104. 10.1126/science.1206025 21596954 PMC3303606

[B27] Felice V. D. Quigley E. M. Sullivan A. M. O’Keeffe G. W. O’Mahony S. M. (2016). Microbiota-gut-brain signalling in Parkinson’s disease: implications for non-motor symptoms. Parkinsonism Relat. Disord. 27, 1–8. 10.1016/j.parkreldis.2016.03.012 27013171

[B28] Forsyth C. B. Shannon K. M. Kordower J. H. Voigt R. M. Shaikh M. Jaglin J. A.. (2011). Increased intestinal permeability correlates with sigmoid mucosa alpha-synuclein staining and endotoxin exposure markers in early Parkinson’s disease. PLoS One 6:e28032. 10.1371/journal.pone.0028032 22145021 PMC3228722

[B29] Goetz C. G. Poewe W. Rascol O. Sampaio C. Stebbins G. T. Counsell C.. (2004). Movement disorder society task force report on the hoehn and yahr staging scale: status and recommendations. Mov. Disord. 19, 1020–1028. 10.1002/mds.20213 15372591

[B30] Haas B. J. Gevers D. Earl A. M. Feldgarden M. Ward D. V. Giannoukos G.. (2011). Chimeric 16S rRNA sequence formation and detection in Sanger and 454-pyrosequenced PCR amplicons. Genome Res. 21, 494–504. 10.1101/gr.112730.110 21212162 PMC3044863

[B31] Hasegawa S. Goto S. Tsuji H. Okuno T. Asahara T. Nomoto K.. (2015). Intestinal dysbiosis and lowered serum lipopolysaccharide-binding protein in Parkinson’s disease. PLoS One 10:e0142164. 10.1371/journal.pone.0142164 26539989 PMC4634857

[B32] Hemmerle A. M. Herman J. P. Seroogy K. B. (2012). Stress, depression and Parkinson’s disease. Exp. Neurol. 233, 79–86. 10.1016/j.expneurol.2011.09.035 22001159 PMC3268878

[B33] Hill-Burns E. M. Debelius J. W. Morton J. T. Wissemann W. T. Lewis M. R. Wallen Z. D.. (2017). Parkinson’s disease and Parkinson’s disease medications have distinct signatures of the gut microbiome. Mov. Disord. 32, 739–749. 10.1002/mds.26942 28195358 PMC5469442

[B34] Hooper L. V. Littman D. R. Macpherson A. J. (2012). Interactions between the microbiota and the immune system. Science 336, 1268–1273. 10.1126/science.1223490 22674334 PMC4420145

[B35] Hopfner F. Kunstner A. Muller S. H. Kunzel S. Zeuner K. E. Margraf N. G.. (2017). Gut microbiota in Parkinson disease in a northern German cohort. Brain Res. 1667, 41–45. 10.1016/j.brainres.2017.04.019 28506555

[B36] Kang D. W. Park J. G. Ilhan Z. E. Wallstrom G. Labaer J. Adams J. B.. (2013). Reduced incidence of Prevotella and other fermenters in intestinal microflora of autistic children. PLoS One 8:e68322. 10.1371/journal.pone.0068322 23844187 PMC3700858

[B37] Keshavarzian A. Green S. J. Engen P. A. Voigt R. M. Naqib A. Forsyth C. B.. (2015). Colonic bacterial composition in Parkinson’s disease. Mov. Disord. 30, 1351–1360. 10.1002/mds.26307 26179554

[B38] Lee Y. K. Menezes J. S. Umesaki Y. Mazmanian S. K. (2011). Proinflammatory T-cell responses to gut microbiota promote experimental autoimmune encephalomyelitis. Proc. Natl. Acad. Sci. U S A 108, 4615–4622. 10.1073/pnas.1000082107 20660719 PMC3063590

[B40] Li W. Wu X. Hu X. Wang T. Liang S. Duan Y.. (2017). Structural changes of gut microbiota in Parkinson’s disease and its correlation with clinical features. Sci. China Life Sci. 60, 1223–1233. 10.1007/s11427-016-9001-4 28536926

[B39] Li B. Zhang X. Guo F. Wu W. Zhang T. (2013). Characterization of tetracycline resistant bacterial community in saline activated sludge using batch stress incubation with high-throughput sequencing analysis. Water Res. 47, 4207–4216. 10.1016/j.watres.2013.04.021 23764571

[B41] Lin A. Zheng W. He Y. Tang W. Wei X. He R.. (2018). Gut microbiota in patients with Parkinson’s disease in southern China. Parkinsonism Relat. Disord. 53, 82–88. 10.1016/j.parkreldis.2018.05.007 29776865

[B44] Lozupone C. A. Hamady M. Kelley S. T. Knight R. (2007). Quantitative and qualitative β diversity measures lead to different insights into factors that structure microbial communities. Appl. Environ. Microbiol. 73, 1576–1585. 10.1128/aem.01996-06 17220268 PMC1828774

[B42] Lozupone C. Knight R. (2005). UniFrac: a new phylogenetic method for comparing microbial communities. Appl. Environ. Microbiol. 71, 8228–8235. 10.1128/aem.71.12.8228-8235.2005 16332807 PMC1317376

[B43] Lozupone C. Lladser M. E. Knights D. Stombaugh J. Knight R. (2011). UniFrac: an effective distance metric for microbial community comparison. ISME J. 5, 169–172. 10.1038/ismej.2010.133 20827291 PMC3105689

[B45] Lozupone C. A. Rhodes M. E. Neff C. P. Fontenot A. P. Campbell T. B. Palmer B. E. (2014). HIV-induced alteration in gut microbiota: driving factors, consequences and effects of antiretroviral therapy. Gut Microbes 5, 562–570. 10.4161/gmic.32132 25078714

[B46] Lundberg D. S. Yourstone S. Mieczkowski P. Jones C. D. Dangl J. L. (2013). Practical innovations for high-throughput amplicon sequencing. Nat. Methods 10, 999–1002. 10.1038/nmeth.2634 23995388

[B47] Magoč T. Salzberg S. L. (2011). FLASH: fast length adjustment of short reads to improve genome assemblies. Bioinformatics 27, 2957–2963. 10.1093/bioinformatics/btr507 21903629 PMC3198573

[B48] Mancabelli L. Milani C. Lugli G. A. Turroni F. Mangifesta M. Viappiani A.. (2017). Unveiling the gut microbiota composition and functionality associated with constipation through metagenomic analyses. Sci. Rep. 7:9879. 10.1038/s41598-017-10663-w 28852182 PMC5575163

[B49] Mayer E. A. Knight R. Mazmanian S. K. Cryan J. F. Tillisch K. (2014). Gut microbes and the brain: paradigm shift in neuroscience. J. Neurosci. 34, 15490–15496. 10.1523/jneurosci.3299-14.2014 25392516 PMC4228144

[B50] Minter M. R. Hinterleitner R. Meisel M. Zhang C. Leone V. Zhang X.. (2017). Antibiotic-induced perturbations in microbial diversity during post-natal development alters amyloid pathology in an aged APP_SWE_/PS1_ΔE9_ murine model of Alzheimer’s disease. Sci. Rep. 7:10411. 10.1038/s41598-017-11047-w 28874832 PMC5585265

[B51] Movement Disorder Society Task Force on Rating Scales for Parkinson’s Disease. (2003). The unified Parkinson’s disease rating scale (UPDRS): status and recommendations. Mov. Disord. 18, 738–750. 10.1002/mds.10473 12815652

[B52] Mulak A. Bonaz B. (2015). Brain-gut-microbiota axis in Parkinson’s disease. World J. Gastroenterol. 21, 10609–10620. 10.3748/wjg.v21.i37.10609 26457021 PMC4588083

[B53] O’Reilly F. Finnan F. Allwright S. Smith G. D. Ben-Shlomo Y. (1996). The effects of caring for a spouse with Parkinson’s disease on social, psychological and physical well-being. Br. J. Gen. Pract. 46, 507–512. 8917868 PMC1239744

[B54] Petrov V. A. Saltykova I. V. Zhukova I. A. Alifirova V. M. Zhukova N. G. Dorofeeva Y. B.. (2017). Analysis of gut microbiota in patients with Parkinson’s disease. Bull. Exp. Biol. Med. 162, 734–737. 10.1007/s10517-017-3700-7 28429209

[B55] Postuma R. B. Berg D. Stern M. Poewe W. Olanow C. W. Oertel W.. (2015). MDS clinical diagnostic criteria for Parkinson’s disease. Mov. Disord. 30, 1591–1601. 10.1002/mds.26424 26474316

[B56] Qian Y. Yang X. Xu S. Wu C. Song Y. Qin N.. (2018). Alteration of the fecal microbiota in Chinese patients with Parkinson’s disease. Brain Behav. Immun. 70, 194–202. 10.1016/j.bbi.2018.02.016 29501802

[B57] Quast C. Pruesse E. Yilmaz P. Gerken J. Schweer T. Yarza P.. (2013). The SILVA ribosomal RNA gene database project: improved data processing and web-based tools. Nucleic Acids Res. 41, D590–D596. 10.1093/nar/gks1219 23193283 PMC3531112

[B58] Reichmann H. (2010). Clinical criteria for the diagnosis of Parkinson’s disease. Neurodegener. Dis. 7, 284–290. 10.1159/000314478 20616563

[B59] Rey F. E. Gonzalez M. D. Cheng J. Wu M. Ahern P. P. Gordon J. I. (2013). Metabolic niche of a prominent sulfate-reducing human gut bacterium. Proc. Natl. Acad. Sci. U S A 110, 13582–13587. 10.1073/pnas.1312524110 23898195 PMC3746858

[B60] Sampson T. R. Debelius J. W. Thron T. Janssen S. Shastri G. G. Ilhan Z. E.. (2016). Gut microbiota regulate motor deficits and neuroinflammation in a model of Parkinson’s disease. Cell 167, 1469.e12–1480.e12. 10.1016/j.cell.2016.11.018 27912057 PMC5718049

[B61] Savica R. Carlin J. M. Grossardt B. R. Bower J. H. Ahlskog J. E. Maraganore D. M.. (2009). Medical records documentation of constipation preceding Parkinson disease: a case-control study. Neurology 73, 1752–1758. 10.1212/WNL.0b013e3181c34af5 19933976 PMC2788809

[B62] Scheperjans F. Aho V. Pereira P. A. Koskinen K. Paulin L. Pekkonen E.. (2015). Gut microbiota are related to Parkinson’s disease and clinical phenotype. Mov. Disord. 30, 350–358. 10.1002/mds.26069 25476529

[B63] Scher J. U. Sczesnak A. Longman R. S. Segata N. Ubeda C. Bielski C.. (2013). Expansion of intestinal *Prevotella copri* correlates with enhanced susceptibility to arthritis. Elife 2:e01202. 10.7554/eLife.01202 24192039 PMC3816614

[B64] Segata N. Izard J. Waldron L. Gevers D. Miropolsky L. Garrett W. S.. (2011). Metagenomic biomarker discovery and explanation. Genome Biol. 12:R60. 10.1186/gb-2011-12-6-r60 21702898 PMC3218848

[B65] Seregin S. S. Golovchenko N. Schaf B. Chen J. Eaton K. A. Chen G. Y. (2017a). NLRP6 function in inflammatory monocytes reduces susceptibility to chemically induced intestinal injury. Mucosal Immunol. 10, 434–445. 10.1038/mi.2016.55 27353251 PMC5199680

[B66] Seregin S. S. Golovchenko N. Schaf B. Chen J. Pudlo N. A. Mitchell J.. (2017b). NLRP6 protects Il10^−/−^ mice from colitis by limiting colonization of akkermansia muciniphila. Cell Rep. 19, 733–745. 10.1016/j.celrep.2017.03.080 28445725 PMC5528001

[B67] Severance E. G. Yolken R. H. Eaton W. W. (2016). Autoimmune diseases, gastrointestinal disorders and the microbiome in schizophrenia: more than a gut feeling. Schizophr. Res. 176, 23–35. 10.1016/j.schres.2014.06.027 25034760 PMC4294997

[B68] Shannon K. M. Keshavarzian A. Dodiya H. B. Jakate S. Kordower J. H. (2012). Is alpha-synuclein in the colon a biomarker for premotor Parkinson’s disease? Evidence from 3 cases. Mov. Disord. 27, 716–719. 10.1002/mds.25020 22550057

[B69] Shen S. S. Shen Y. Xiong K. P. Chen J. Mao C. J. Huang J. Y.. (2014). Validation study of REM sleep behavior disorder questionnaire-Hong Kong (RBDQ-HK) in east China. Sleep Med. 15, 952–958. 10.1016/j.sleep.2014.03.020 24938584

[B70] Smits S. A. Leach J. Sonnenburg E. D. Gonzalez C. G. Lichtman J. S. Reid G.. (2017). Seasonal cycling in the gut microbiome of the Hadza hunter-gatherers of Tanzania. Science 357, 802–806. 10.1126/science.aan4834 28839072 PMC5891123

[B71] Sonnenburg J. L. Bäckhed F. (2016). Diet-microbiota interactions as moderators of human metabolism. Nature 535, 56–64. 10.1038/nature18846 27383980 PMC5991619

[B72] Unger M. M. Spiegel J. Dillmann K. U. Grundmann D. Philippeit H. Burmann J.. (2016). Short chain fatty acids and gut microbiota differ between patients with Parkinson’s disease and age-matched controls. Parkinsonism Relat. Disord. 32, 66–72. 10.1016/j.parkreldis.2016.08.019 27591074

[B73] Visser M. Marinus J. Stiggelbout A. M. Van Hilten J. J. (2004). Assessment of autonomic dysfunction in Parkinson’s disease: the SCOPA-AUT. Mov. Disord. 19, 1306–1312. 10.1002/mds.20153 15390007

[B74] Wang Q. Garrity G. M. Tiedje J. M. Cole J. R. (2007). Naive Bayesian classifier for rapid assignment of rRNA sequences into the new bacterial taxonomy. Appl. Environ. Microbiol. 73, 5261–5267. 10.1128/aem.00062-07 17586664 PMC1950982

[B75] White J. R. Nagarajan N. Pop M. (2009). Statistical methods for detecting differentially abundant features in clinical metagenomic samples. PLoS Comput. Biol. 5:e1000352. 10.1371/journal.pcbi.1000352 19360128 PMC2661018

[B76] Yatsunenko T. Rey F. E. Manary M. J. Trehan I. Dominguez-Bello M. G. Contreras M.. (2012). Human gut microbiome viewed across age and geography. Nature 486, 222–227. 10.1038/nature11053 22699611 PMC3376388

[B77] Zapala M. A. Schork N. J. (2006). Multivariate regression analysis of distance matrices for testing associations between gene expression patterns and related variables. Proc. Natl. Acad. Sci. U S A 103, 19430–19435. 10.1073/pnas.0609333103 17146048 PMC1748243

[B78] Zheng P. Zeng B. Zhou C. Liu M. Fang Z. Xu X.. (2016). Gut microbiome remodeling induces depressive-like behaviors through a pathway mediated by the host’s metabolism. Mol. Psychiatry 21, 786–796. 10.1038/mp.2016.44 27067014

[B79] Zhu L. Liu W. Alkhouri R. Baker R. D. Bard J. E. Quigley E. M.. (2014). Structural changes in the gut microbiome of constipated patients. Physiol. Genomics 46, 679–686. 10.1152/physiolgenomics.00082.2014 25073603

