# Supplementary material for: Gut Microbiota Differs Between Parkinson’s Disease Patients and Healthy Controls in Northeast China
Source: Front Mol Neurosci. 2019 Jul 11;12:171. doi: 10.3389/fnmol.2019.00171 (PMC6637281; doi:10.3389/fnmol.2019.00171)
Supplement: Supplementary file 1 [file Presentation_1.pdf]

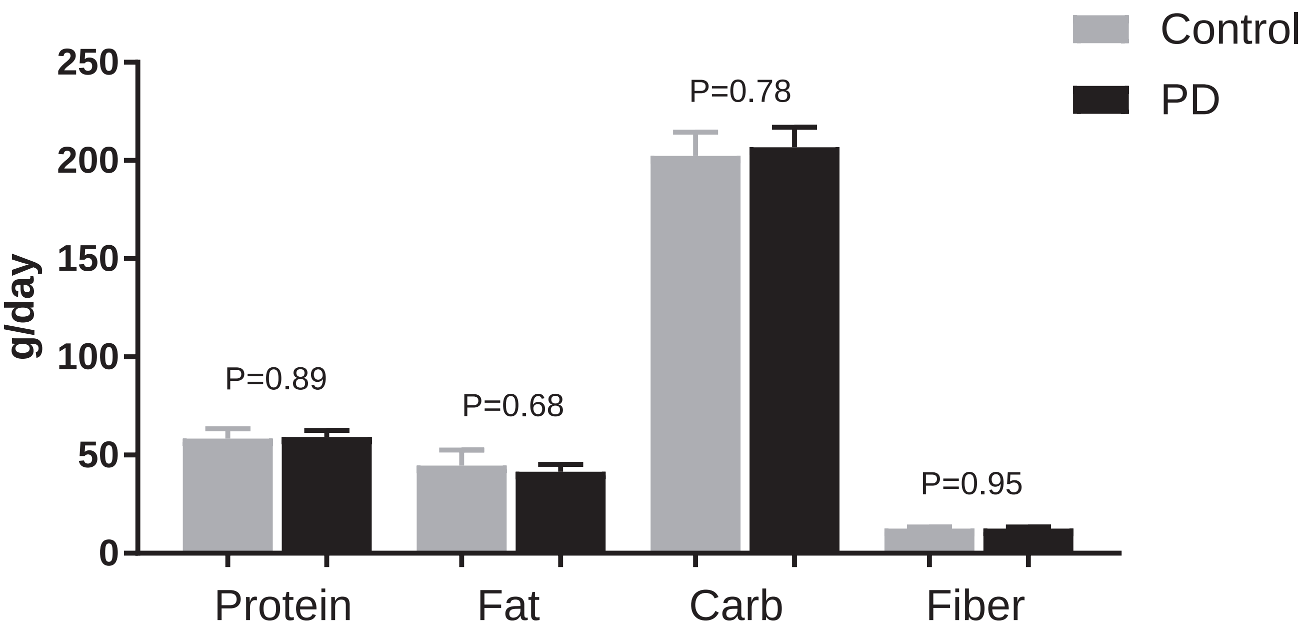

Supplemental Figure 1. Comparison of dietary intake between PD patients and controls. Unpaired t-test, n=42 for PD, n=23 for control. Data are presented as mean ± s.e.m.

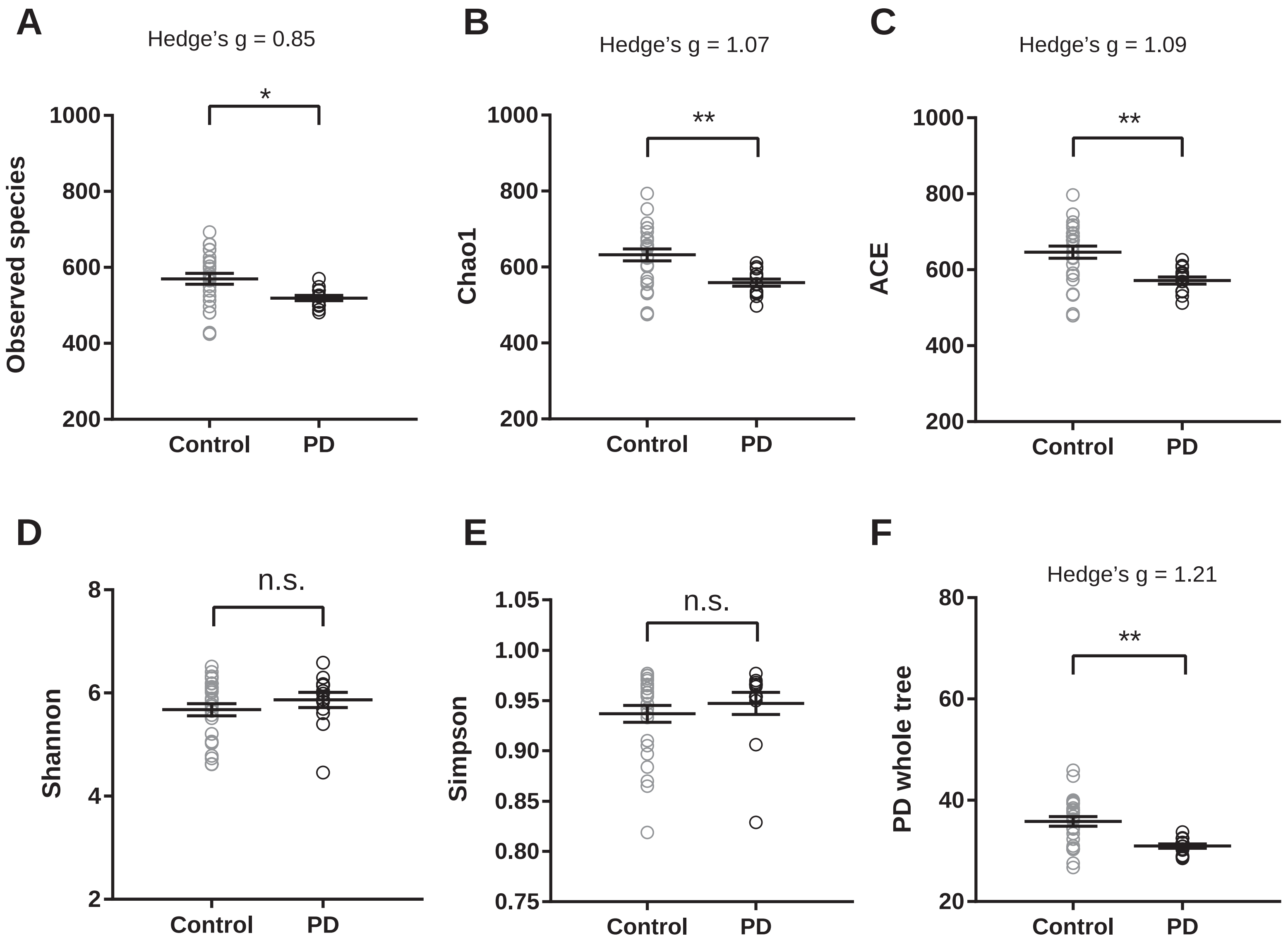

Supplemental Figure 2. Comparison of microbiota richness and diversity indices between PD patients and controls with constipation Wexner score ≤ 3. A-C: species richness, D-E: α-diversity, F: phylogenetic diversity. n=13 for PD, n=25 for control. Data are presented as mean±s.e.m. with individual replicates also shown. *p<0.05, **p<0.01, n.s.: not significant, unpaired t-test.

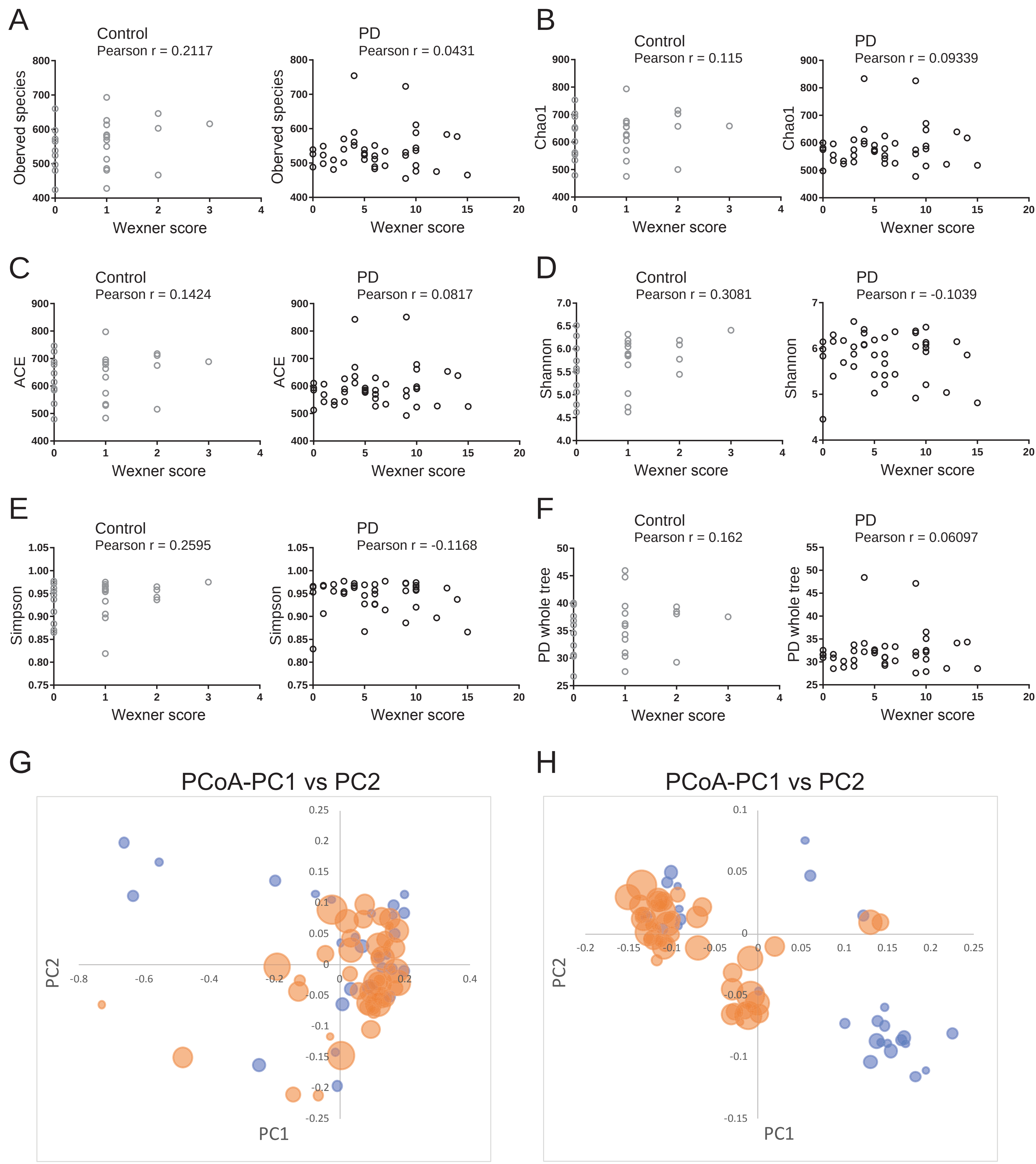

Supplemental Figure 3. Impact of constipation on gut microbiota richness and diversity indices. A-F: Correlation analysis to assess the impact of constipation on species richness, α-diversity and phylogenetic diverstiy indices. G,H: Impact of constipation on β-diversity. Data shown are the results of Principal Co-ordinates Analysis (PCoA) plotted with weighted Unifrac (G) and unweighted Unifrac (H) in controls (blue bubbles) and PD patients (orange bubbles). The size of the bubbles indicates the severity of the constipation (Wexner score).

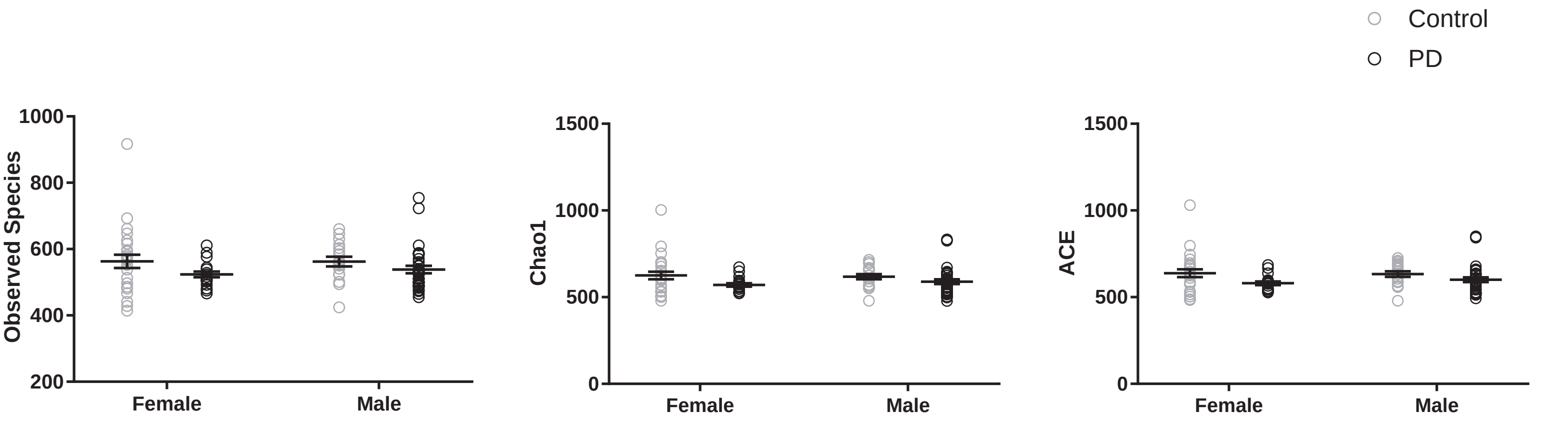

Two-way ANOVA:
Interaction P=0.6267
Main effect 1 (Gender) P=0.6512
Main effect 2 (PD) **P=0.0436**

Two-way ANOVA:
Interaction P=0.4595
Main effect 1 (Gender) P=0.7348
Main effect 2 (PD) **P=0.0181**

Two-way ANOVA:
Interaction P=0.4748
Main effect 1 (Gender) P=0.6693
Main effect 2 (PD) **P=0.0131**

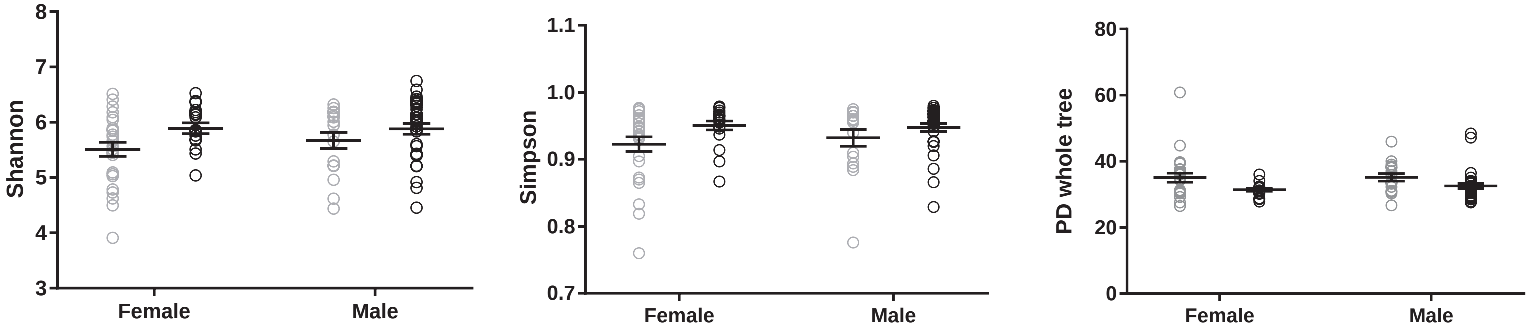

Two-way ANOVA:
Interaction P=0.4805
Main effect 1 (Gender) P=0.5196
Main effect 2 (PD) **P=0.0162**

Two-way ANOVA:
Interaction P=0.5063
Main effect 1 (Gender) P=0.7291
Main effect 2 (PD) **P=0.0215**

Two-way ANOVA:
Interaction P=0.6097
Main effect 1 (Gender) P=0.5503
Main effect 2 (PD) **P=0.0042**

Supplemental Figure 4. Two-way ANOVA analysis to assess the impact of gender and PD on species richness, α-diversity and phylogenetic diverstiy indices.

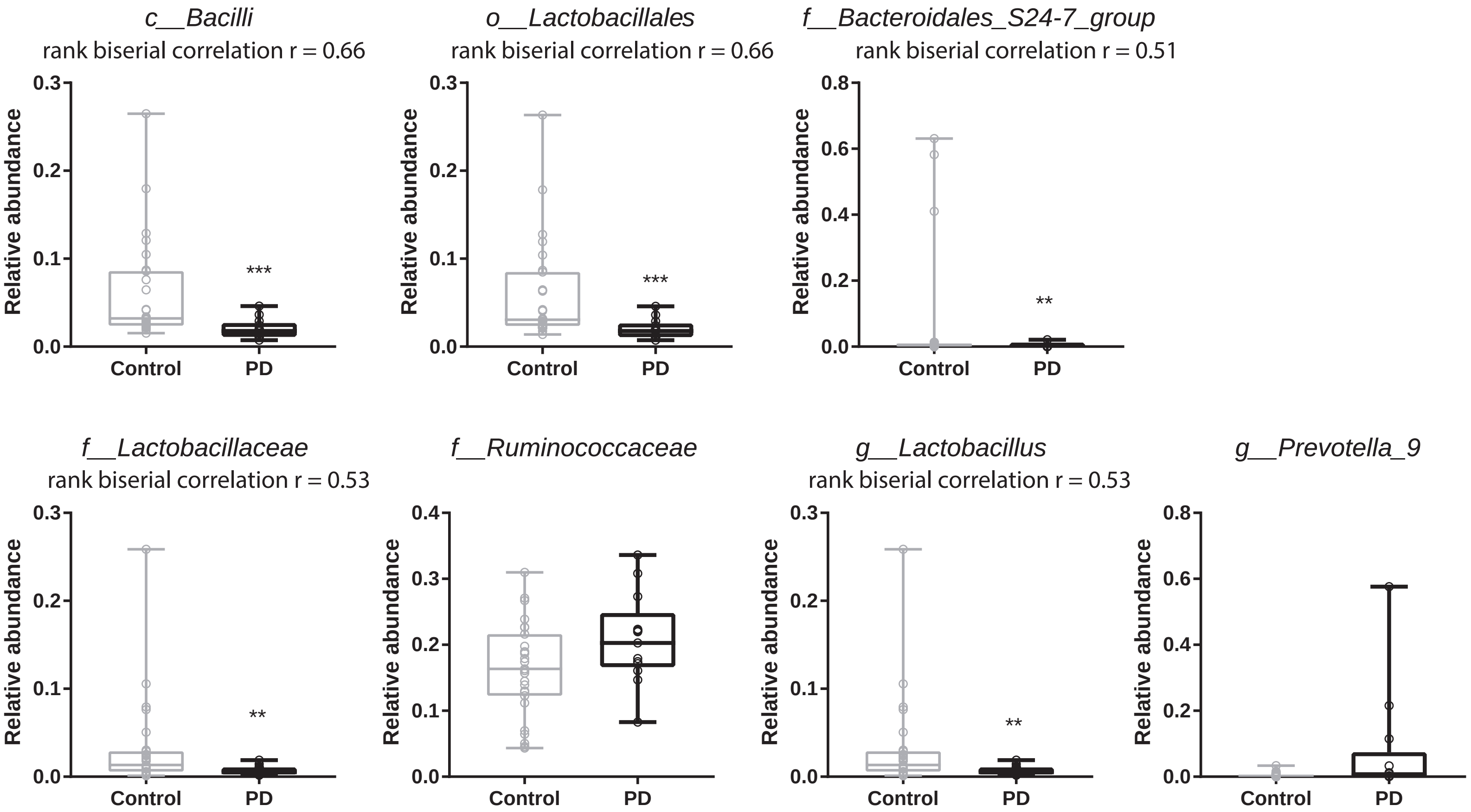

Supplemental Figure 5. Comparison of microbiota abundances between PD patients and controls with constipation Wexner score ≤ 3. Wilcoxon rank-sum test, n=13 for PD, n=25 for control. **p<0.01, ***p<0.001, .

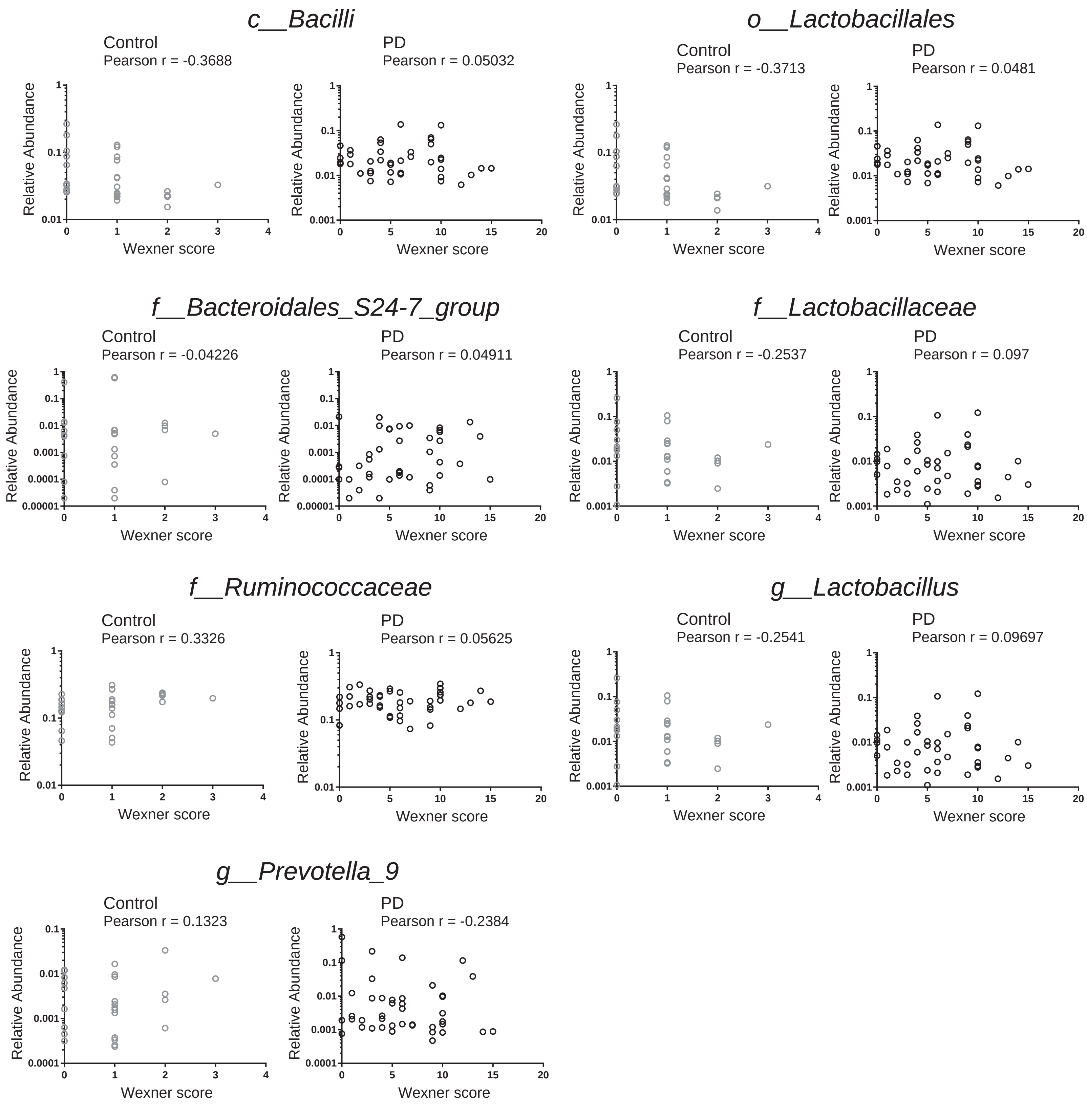

Supplemental Figure 6. Correlation analysis to assess the impact of constipation on the relative abundance of individual taxa. The correlations between the abundances and the Wexner constipation scores are weak, suggesting that the severity of constipation does not have a strong effect on the abundances of these taxa.

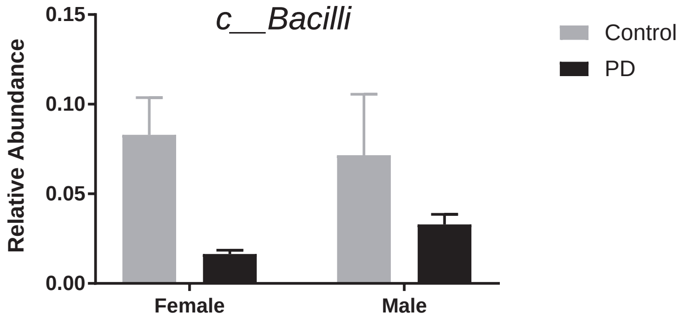

ANOVA table	SS (Type III)	DF	MS	F (DFn, DFd)	P value
Interaction	0.004273	1	0.004273	F (1, 90) = 0.6149	P=0.4350
Sex	0.0001383	1	0.0001383	F (1, 90) = 0.0199	P=0.8881
PD vs control	0.06107	1	0.06107	F (1, 90) = 8.79	**P=0.0039**
Residual	0.6253	90	0.006948		

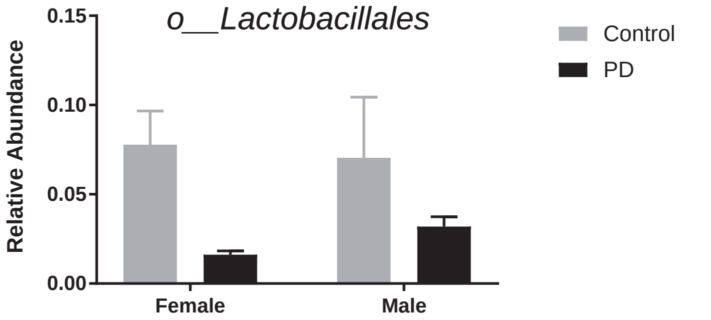

ANOVA table	SS (Type III)	DF	MS	F (DFn, DFd)	P value
Interaction	0.002921	1	0.002921	F (1, 90) = 0.4577	P=0.5004
Sex	0.0003813	1	0.0003813	F (1, 90) = 0.05975	P=0.8074
PD vs control	0.05545	1	0.05545	F (1, 90) = 8.689	**P=0.0041**
Residual	0.5743	90	0.006381		

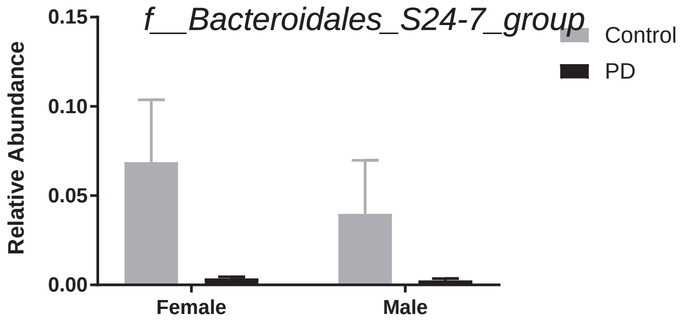

ANOVA table	SS (Type III)	DF	MS	F (DFn, DFd)	P value
Interaction	0.004315	1	0.004315	F (1, 90) = 0.3744	P=0.5422
Sex	0.004997	1	0.004997	F (1, 90) = 0.4336	P=0.5119
PD vs control	0.05778	1	0.05778	F (1, 90) = 5.013	**P=0.0276**
Residual	1.037	90	0.01152		

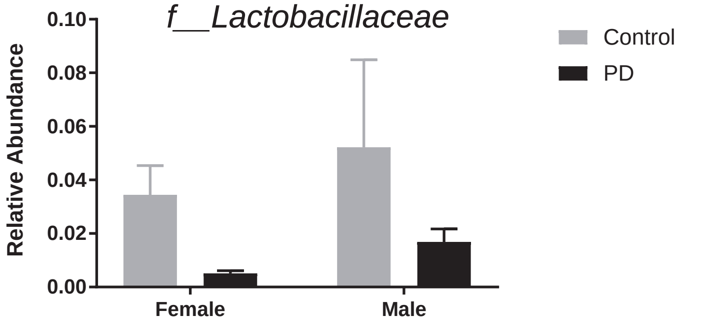

ANOVA table	SS (Type III)	DF	MS	F (DFn, DFd)	P value
Interaction	0.0001958	1	0.0001958	F (1, 90) = 0.045	P=0.8325
Sex	0.004801	1	0.004801	F (1, 90) = 1.103	P=0.2964
PD vs control	0.02309	1	0.02309	F (1, 90) = 5.306	**P=0.0236**
Residual	0.3917	90	0.004352		

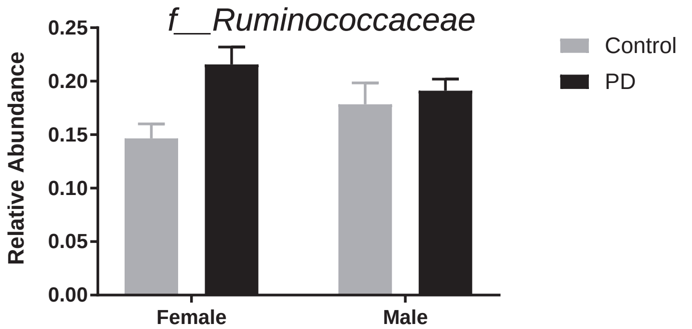

ANOVA table	SS (Type III)	DF	MS	F (DFn, DFd)	P value
Interaction	0.01754	1	0.01754	F (1, 90) = 3.639	P=0.0596
Sex	0.0003045	1	0.0003045	F (1, 90) = 0.0632	P=0.8021
PD vs control	0.03713	1	0.03713	F (1, 90) = 7.707	**P=0.0067**
Residual	0.4336	90	0.004818		

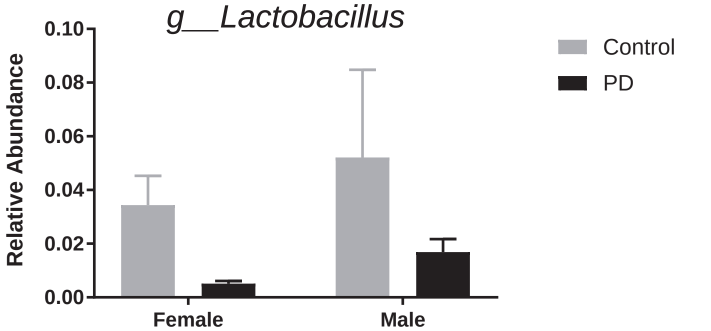

ANOVA table	SS (Type III)	DF	MS	F (DFn, DFd)	P value
Interaction	0.0002024	1	0.0002024	F (1, 90) = 0.04656	P=0.8297
Sex	0.004815	1	0.004815	F (1, 90) = 1.108	P=0.2954
PD vs control	0.02301	1	0.02301	F (1, 90) = 5.293	**P=0.0237**
Residual	0.3913	90	0.004348		

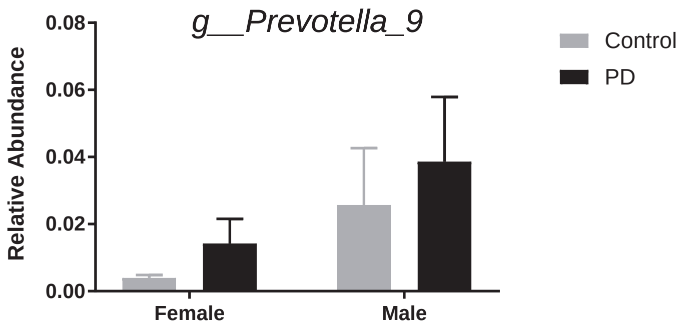

ANOVA table	SS (Type III)	DF	MS	F (DFn, DFd)	P value
Interaction	0.00003773	1	3.773E-05	F (1, 90) = 0.007314	P=0.9320
Sex	0.01176	1	0.01176	F (1, 90) = 2.279	P=0.1347
PD vs control	0.002972	1	0.002972	F (1, 90) = 0.576	P=0.4498
Residual	0.4643	90	0.005159		

Supplemental Figure 7. Two-way ANOVA analysis to assess the impact of gender and PD on gut microbiota taxa identified by LDA scores>4 in LEfSe analysis.

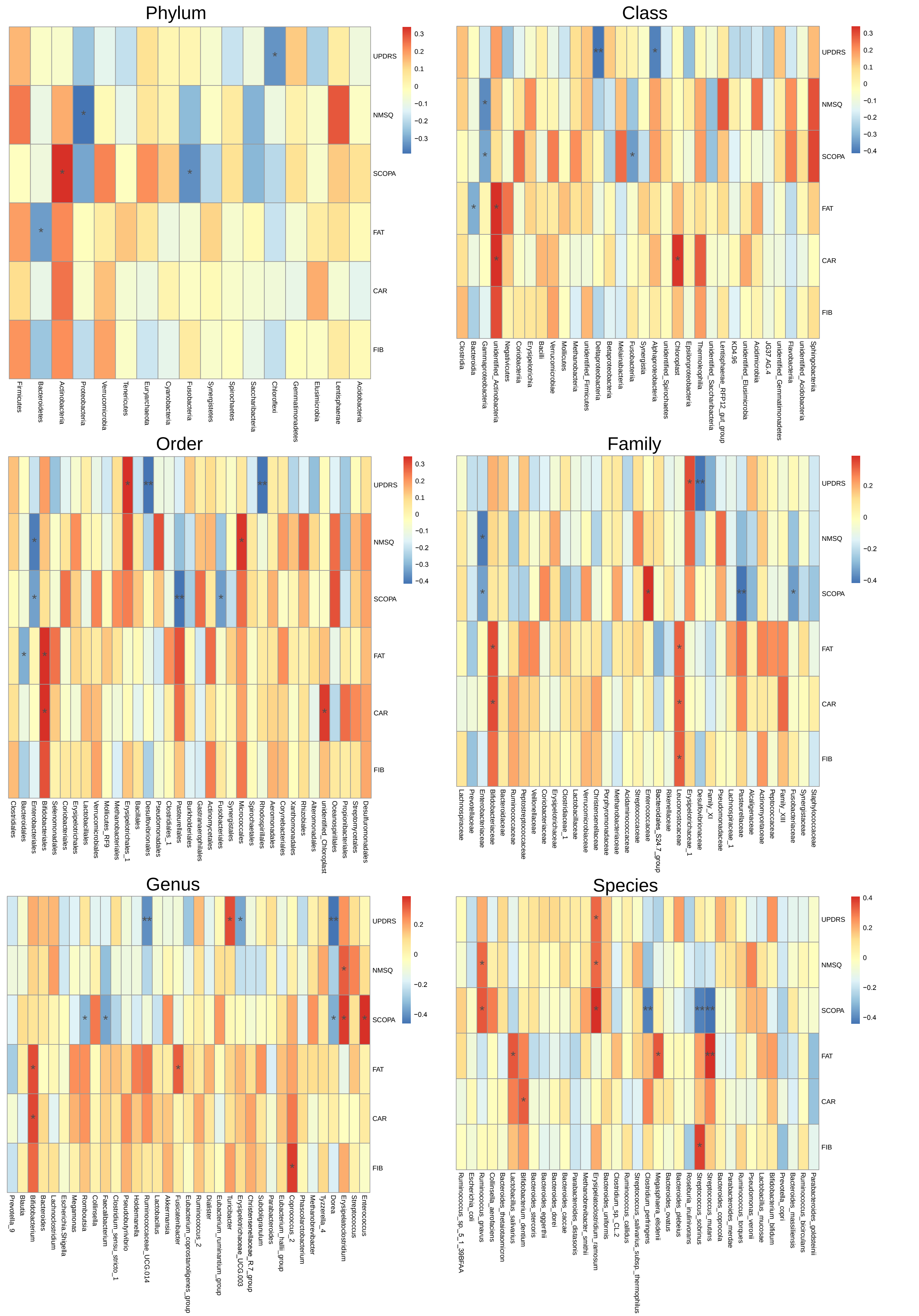

Supplemental Figure 8. Correlations between environmental factors and gut microbiota in PD patients. Correlation heatmaps showing the relationship between environmental factors and gut microbiota at different taxonomic ranks. Each row in the heatmap represents an environmental factor. Each column represents a taxon. The color temperature encodes Spearman correlation coefficient r. n=42, *p < 0.05, ** p < 0.01.

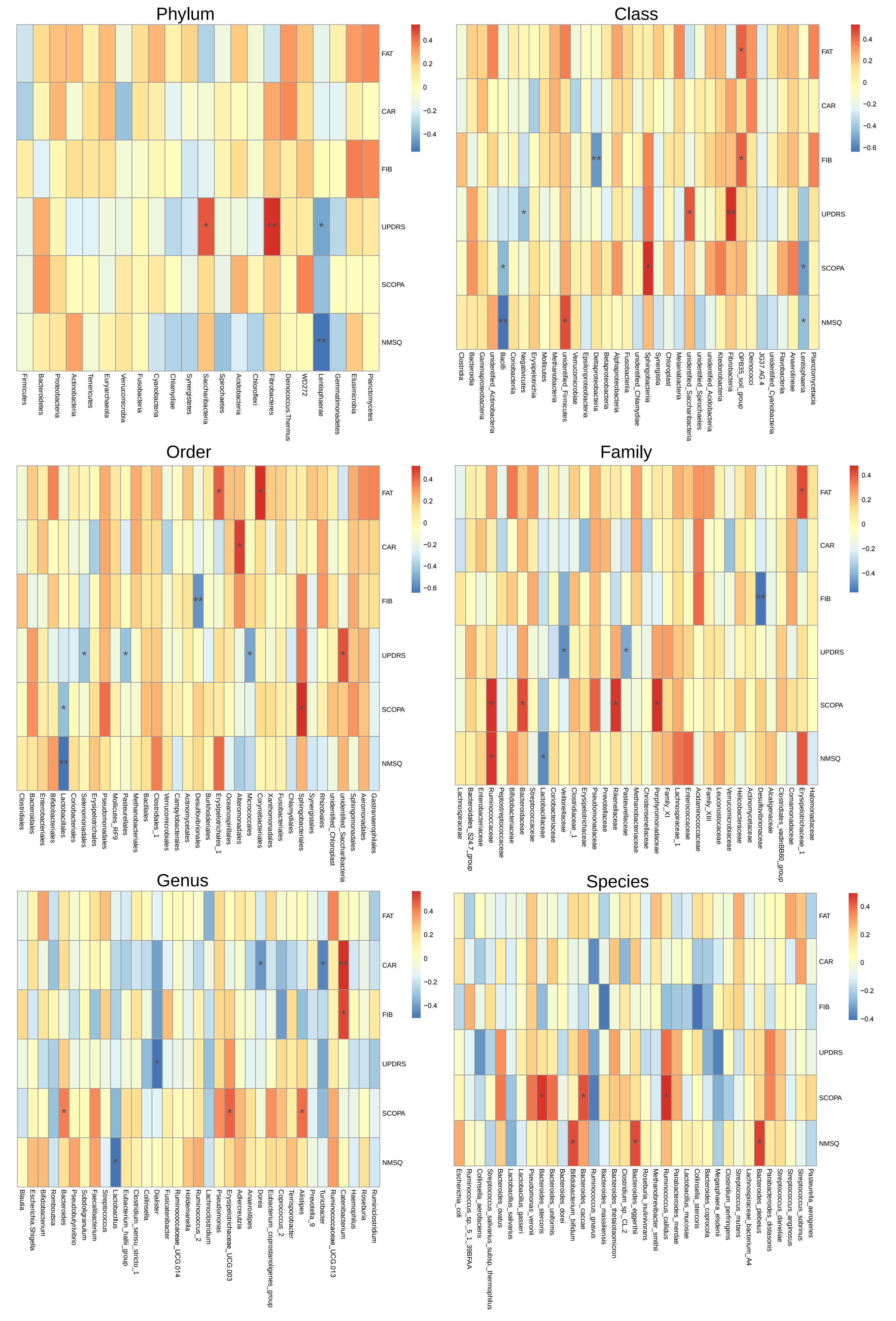

Supplemental Figure 9. Correlations between clinical and dietary factors and gut microbiota in healthy controls. Correlation heatmaps showing the relationship between clinical and dietary factors and gut microbiota at different taxonomic ranks. Each row in the heatmap represents a clinical or dietary factor. Each column represents a taxon. The color temperature encodes Spearman correlation coefficient r. n=23, *p < 0.05, ** p < 0.01.
